# Microcirculatory responses at rest and during exercise in limited cutaneous systemic sclerosis compared to age-matched healthy adults

**DOI:** 10.3389/fspor.2026.1872867

**Published:** 2026-08-11

**Authors:** Alexandros Mitropoulos, Mohammed Akil, Markos Klonizakis

**Affiliations:** 1 Lifestyle, Exercise and Nutrition Improvement (LENI) Research Group, Centre for Applied Health & Social Care Research (CARe) - School of Health and Social Care College of Health, Wellbeing and Life Sciences, Sheffield Hallam University, Sheffield, United Kingdom; 2 Sheffield Teaching Hospitals NHS Foundation Trust, Rheumatology Department, Royal Hallamshire Hospital, Sheffield, United Kingdom

**Keywords:** digital microcirculation, exercise, iontophoresis, limited cutaneous systemic sclerosis, microvascular function, systemic sclerosis

## Abstract

**Objectives:**

Raynaud's phenomenon (RP) is an early manifestation of systemic sclerosis (SSc), reflecting underlying microvascular dysfunction. While exercise improves vascular function, the magnitude and nature of microvascular impairment relative to healthy physiology remain poorly characterised. This study compared resting digital endothelial-dependent and endothelial-independent microvascular responses, together with systemic microcirculatory responses to exercise, between people with limited cutaneous SSc (lcSSc) and age- and gender-matched healthy adults.

**Methods:**

In this cross-sectional case-control study, thirty-four people with lcSSc (65 ± 12 years; 3 males) and 34 healthy adults (64 ± 11 years; 6 males) were assessed. Microvascular function was evaluated using laser Doppler flowmetry (LDF) with iontophoresis of acetylcholine (ACh; endothelial-dependent) and sodium nitroprusside (SNP; endothelial-independent). Systemic physiological responses to exercise were assessed using transcutaneous oxygen pressure (TcpO₂) was measured from the scapular region during moderate-intensity constant-load exercise. Cutaneous vascular conductance (CVC), maximum response (CVCmax), and time to peak response (Tmax) were analysed.

**Results:**

Significant baseline differences were observed for ACh [0.18 (0.09) vs 0.26 (0.09) lcSSc vs HA; *p* < 0.0001] and SNP [0.19 (0.09) vs 0.27 (0.07); *p* < 0.0001]. Similar findings were observed for CVCmax [e.g., 1.39 (0.83) vs 3.52 (1.26); *p* < 0.0001 for ACh] and TcpO₂. No differences were observed for Tmax.

**Conclusions:**

People with lcSSc exhibit marked impairment in resting digital microvascular function affecting both endothelial-dependent and endothelial-independent pathways, alongside a reduced systemic physiological response to exercise, assessed using scapular TcpO₂. These findings highlight diminished microvascular reserve and provide a basis for developing targeted, individualised dose-dependent exercise interventions.

**Trial registration:**

ClinicalTrials.gov (NCT number): NCT03058887. https://clinicaltrials.gov/ct2/show/NCT03058887?term=NCT03058887&rank=1

## Introduction

Systemic sclerosis (SSc) is a chronic autoimmune rheumatoid condition of unknown origin which is clinically characterized by skin fibrosis extended to internal organs which then lead to altered organ structure and ultimately organ dysfunction. SSc pathophysiology is also expressed by functional and structural vasculopathy leading but not limited to Raynaud phenomenon (RP), digital ulcers (DUs), pulmonary artery hypertension, and renal crisis (1).

RP is the heralding clinical manifestation observed in SSc. This microvasculature disorder (2) affects mainly the fingers and toes. Over 95% of people with SSc (PwSSc) experience RP that precedes to other clinical symptoms observed in SSc. Evidence suggests that RP is triggered by endothelial injuries in association with dysregulations in the production of nitric oxide (NO) and vasoactive factors (2). RP can lead to the development of DUs in people with SSc which are often difficult to heal, threatening thus concomitant infections and in some rare occasions may lead to digital amputations (3).

Exercise interventions have been shown to improve digital microvascular function in PwSSc i alongside reductions in the frequency and severity of RP attacks, suggesting a potential role in mitigating vascular complications such as DUs (4, 5). Nevertheless, the physiological basis for optimising such interventions remains poorly understood. In particular, the magnitude and nature of microvascular impairment relative to healthy physiological function have not been clearly defined.

This represents an important gap, as the effectiveness of exercise as a therapeutic strategy is inherently dose-dependent (6, 7) and influenced by baseline vascular function. Moreover, there is increasing recognition that individualised exercise prescription is more effective than generalised approaches, particularly in clinical populations with heterogeneous pathophysiology (8). In this context, a precise understanding of the underlying microvascular impairment is essential to inform tailored intervention strategies. Without a clear characterisation of the extent to which endothelial-dependent and endothelial-independent pathways are impaired in SSc, it remains difficult to determine the optimal intensity, duration, or type of stimulus required to elicit meaningful vascular adaptations.

Previous studies using laser Doppler flowmetry and iontophoresis have demonstrated impaired endothelial-dependent and endothelial-independent vasodilatory responses in SSc (9–11). However, these findings have not been consistently interpreted in relation to the magnitude of deviation from healthy physiological function, nor extended to assess responses under physiological stress conditions relevant to intervention design.

Furthermore, it remains unclear whether the microcirculation in SSc is capable of appropriately responding to physiological stress, such as exercise-induced hyperaemia, which is a key mechanism underpinning vascular adaptation.

Therefore, characterising microvascular responses both at rest and during physiological challenge, and benchmarking these against healthy individuals, is a necessary step toward informing the design and optimisation of targeted interventions aimed at improving vascular function in SSc. The present study combines assessment of resting digital microvascular function with assessment of systemic physiological responses to exercise using scapular TcpO₂, providing complementary information regarding vascular function at rest and during physiological stress.

This study aimed to quantify the magnitude of digital microvascular impairment at rest in limited cutaneous (lcSSc) relative to healthy physiological function, and to assess its responsiveness under systemic physiological stress (during exercise), in order to provide a mechanistic basis for the development and optimisation of targeted interventions such as exercise.

## Methods

### Study design

This was a cross-sectional, case–control study comparing microvascular function between people with lcSSc and age- and gender-matched healthy controls.

### Participants

We recruited 34 (31 women, 3 men; *self-defined gender*) people with lcSSc, defined as per the American College of Rheumatology and European league against rheumatism criteria (12). We also recruited 34 (28 women, 6 men; *self-defined gender*) healthy adults. Participants with lcSSc were required to be on stable medical therapy, including vasoactive medication where prescribed, for at least 8 weeks prior to participation. No changes in medication were permitted during this period. Healthy controls were matched to the lcSSc group at the group level according to age and sex. Individual one-to-one matching was not performed. Healthy adults were defined as free from any rheumatic or cardiovascular disease, who were not receiving any regular medication expected to influence vascular function, were not pregnant, and were leading a sedentary lifestyle. Eligible people with lcSSc were recruited from the Rheumatology Department of the Royal Hallamshire Hospital in Sheffield. The eligibility criteria for people with lcSSc are presented in Table 1. All participants provided written consent to participate. The London - West London & GTAC NHS Research Ethics Committee approved the study protocol and the study complies with the Declaration of Helsinki. Our study was registered to ClinicalTrials.gov (NCT number): NCT03058887.

**Table 1:** Eligibility criteria for people with lcSSc.

Inclusion criteria	Exclusion criteria
Patients diagnosed with Limited Cutaneous Systemic Sclerosis according to the 2013 ACR/EULAR criteria experiencing Raynaud's phenomenon.	Patients with advanced pulmonary arterial hypertension or interstitial lung disease.
Men or women aged ≥18 years old.	Patients who are diagnosed with another inflammatory condition.
Disease duration between 1 to 10 years.	Patients presenting myositis with proximal muscle weakness.
Patients should be able to perform exercise.	Patients with New York Heart Association class 3 or 4.
	Current smokers or people who stopped smoking within 4 weeks of health screening.
	Women who are currently pregnant.

ACR, American College of Rheumatology; EULAR, European League Against Rheumatism.

Participants were restricted to individuals with lcSSc to reduce disease heterogeneity and allow for a more focused assessment of microvascular function. lcSSc represents a more clinically homogeneous subgroup, with relatively less severe systemic involvement compared to diffuse disease (3, 13), thereby minimising potential confounding from advanced organ pathology. This approach enables clearer interpretation of microvascular responsiveness and its relevance to physiological function and intervention design.

All enrolled participants completed the study protocol. There were no post-enrolment exclusions, missing outcome data or participant drop-outs, and all participants were included in the final analyses.

### Procedures

Participants were requested to perform a single visit at Sheffield Hallam University (Collegiate Campus, Sheffield, UK). The assessments for all participants included anthropometry and microvascular reactivity using two non-invasive vascular assessment methods i) laser Doppler Flowmetry (LDF) coupled with iontophoresis at rest and ii) systemic physiological responses to exercise using transcutaneous oxygen pressure (TcpO₂) measured at the scapular region during continuous moderate-intensity exercise. Participants were instructed to abstain from caffeine (12 h), nicotine and alcohol (24 h), and vigorous exercise (24 h) prior to testing. All assessments were conducted in a temperature-controlled laboratory (22°C - 24°C) following an acclimatisation period of 10-15 minutes. The microvascular assessment using LDF coupled with iontophoresis was completed before the exercise protocol incorporating TcpO₂ measurements. Following completion of the vascular assessment, participants were allowed an appropriate recovery period before commencing the exercise protocol to minimise any potential carry-over effects.

## Study outcomes

### Anthropometry

The participants’ stature (cm) was measured using a Hite-Rite Precision Mechanical Stadiometer. Body weight (kg), body mass index (BMI), fat mass (kg) and lean body mass (kg) segmented in upper and lower-limbs were assessed by using bio-electrical impedance analysis (In Body 720, Seoul, Korea). Participants’ demographic characteristics are illustrated in Table 2.

**Table 2:** Demographic data (means ± SD).

Demographics	People with lcSSc (*n* = 34)	Healthy adults (*n* = 34)	*P*-value
Age (years)	65 ± 12	64 ± 12	0.73
Gender (male)	3	6	0.48
Body weight (kg)	70 ± 14	64 ± 10	0.047
Soft lean mass (kg)	42 ± 10	40 ± 8	0.37
Fat free mass (kg)	44 ± 9	43 ± 8	0.67
Fat mass (kg)	25 ± 9	23 ± 8	0.34
Body mass index (kg/m^2^)	25.7 ± 4	26.6 ± 5.1	0.42
Stature (cm)	163 ± 8	160 ± 7	0.10
Disease duration (years)	8 ± 2	N/A	
Medications (average)	5 ± 2	1 ± 1	
Resting systolic blood pressure (mmHg)	131 ± 17	124 ± 6	
Resting diastolic blood pressure (mmHg)	80 ± 9	77 ± 8	

### Microvascular reactivity

Microvascular function was assessed with the use of LDF coupled with Iontophoresis in a temperature-controlled room (23-25°C). LDF electrodes were attached to the dorsal aspect of the reference fingers for acetylcholine (ACh) and sodium nitroprusside (SNP) administration. ACh and SNP were used as indicators of the changes occurring in the digital endothelial –dependent and –independent vasodilatory function. The results are presented as cutaneous vascular conductance (CVC) to account for the variability of blood pressure during the assessment.

To calculate CVC and monitor participant wellbeing, heart rate (Sports Tester, Polar, Finland) and blood pressure of the brachial artery (left arm; Dinamap Dash 2500, GE Healthcare, USA) were monitored at 5-minute intervals throughout the study period. The two drug delivery electrodes (PF383; Perimed AB, Jarfalla, Sweden) were positioned over healthy looking skin, approximately 4 cm apart. One was containing 100 μL of 1% ACh (Miochol-E, Novartis, Stein) and the other 80 μl of 1% SNP (Nitroprussiat, Rottapharm). ACh was placed over the middle finger between the distal and proximal interphalangeal joints and SNP was placed over the index finger between the metacarpophalangeal and carpometacarpal joints. The incremental iontophoresis protocol for ACh and SNP delivery is described in detail Klonizakis et al. (14, 15).

The time to peak vascular response (T_max_) is also reported. T_max_ is a complimentary microcirculatory measure, focusing on vascular stiffness. Its reproducibility has been assessed elsewhere (16).

### Transcutaneous oxygen pressure (TcpO_2_) and cycle ergometry test

TcpO_2_ measurements were performed during a cycle ergometry exercise test with constant load. Participants performed a constant-load cycling protocol (cycle ergometer, Monark 894E, Vansbro, Sweden) with cadence maintained at approximately 65 rpm. During the first 5 minutes, workload was individually adjusted to achieve a perceived exertion of approximately 13 (“somewhat hard”) on the Borg scale (6-20) (17), corresponding to a moderate exercise intensity, which was maintained throughout the exercise period. Heart rate (HR) was recorded throughout the exercise protocol. Participants with lcSSc achieved a mean HR of 110 ± 5 bpm, while healthy adults achieved a mean HR of 114 ± 4 bpm. Participants then completed approximately 20 minutes of constant-load exercise followed by 5 minutes of unloaded recovery. Exercise early termination criteria were upon participant request or if musculoskeletal pain or fatigue prevented continuation. The exercise protocol was designed to provide a standardised moderate-intensity physiological stimulus rather than assess exercise performance. Therefore, absolute workload (W) was not recorded, as the objective was to standardise the internal physiological stimulus rather than the external mechanical work performed (17). TcpO_2_ sensors that were non-invasively attached onto the skin and allowed to heat were used. The sensors induce skin blood capillaries dilatation through heat, which increases the blood flow and results in oxygen diffusion through the skin to the sensor. The sensor measures TcpO_2_ values inwardly through an electrochemical process.

Measurements were performed using the TINA TCM400 TcpO_2_ device (Radiometer, Copenhagen, Denmark) following a 20 min resting period. The room temperature was set at 21 ± 2°C. The temperature of the probe was set to 44.5°C to allow maximal skin vasodilation, thereby decreasing the arterial to skin surface oxygen pressure gradient. Before the exercise test 15-20 minutes were allowed with the probe attached on the skin for stabilisation of TcpO_2_ values. After the test the TcpO_2_ values were automatically corrected according to a temperature of 37°C by the TINA device. The electrode was placed slightly below the right scapula on the back away from any bone.

Fixation rings were used to hold the probe attached to the skin and this was filled with two small drops of contact fluid before attachment to the sensor. The fluid was then heated causing the subsequent dilatation of the skin. The raw values of the participants’ oxygen perfusion, obtained directly from TcpO_2_ device, were defined as previously described in Wasilewski et al. (18).

## Sample size

The primary outcome for the sample size calculation was the between-group difference in CVC, assessed by LDF coupled with iontophoresis. For our calculations, we used commercial software (G*Power 3.1.7, HHU of Düsseldorf) by including data from a study that examined the microvascular reactivity using LDF coupled with iontophoresis in healthy and diseased individuals (19). Based on those calculations, 34 participants in each group (68 in total) were required to detect between groups differences concerning microvascular reactivity (significance level = 0.05; power =80%).

## Overall data analysis

Data analysis was performed using SPSS software (version 30, IBM SPSS, New York, USA) and is presented as mean ± SD. Normal distribution of the data and homogeneity of variances were tested using the Shapiro–Wilk and Levene's test, respectively. Where assumptions were not met, the appropriate alternative statistical procedures were applied. The comparison between the two groups was performed through independent t-tests and Chi-squared tests. Fisher's exact test was used to compare gender distribution between groups. Effect sizes (Cohen's d) were calculated using the pooled standard deviation for all between-group comparisons, with values of 0.2, 0.5, and 0.8 representing small, medium, and large effects, respectively (20). Statistical significance was set at *p* ≤ 0.05.

Microvascular reactivity assessed by LDF coupled with iontophoresis was the primary outcome and formed the basis of the sample size calculation. TcpO₂ outcomes were considered complementary physiological measures of the microvascular response to exercise-induced physiological stress. As the analyses addressed a limited number of pre-specified outcomes, no formal adjustment for multiple comparisons was performed.

## Results

### Systemic physiological response to exercise (scapular TcpO₂)

Both *Δ*TcpO_2_ [e.g., 3.3 (4.7) vs 5.6 (3.6); *p* < 0.05] and *Δ*TcpO_2max_ [e.g., 10.9 (5.2) vs 13.2 (6.9)] were also higher among the healthy population, in comparison to their lcSSc counterparts (Table 3). Effect sizes were considered medium-to-large (e.g., 0.72 and 0.66 respectively), for both measures.

**Table 3:** Physiological outcomes.

LDF and TcpO2 outcomes	lcSSc (*n* = 34)	Healthy adults (*n* = 34)	*P* value	Effect size (Cohen's d)	95% CI (between-group mean difference)
ACh CVC	0.18 ± 0.09	0.26 ± 0.09	*p* < 0.0001	1.11	0.08 (0.036, 0.124)
ACh CVCmax	1.39 ± 0.83	3.52 ± 1.26	*p* < 0.0001	1.97	2.13 (1.61, 2.65)
ACh Tmax (sec)	152 ± 66	136 ± 59	0.36	0.23	−16 (−46.3, 14.3)
SNP CVC	0.19 ± 0.09	0.27 ± 0.07	*p* < 0.0001	0.99	0.08 (0.04, 0.12)
SNP CVCmax	1.68 ± 1.49	3.22 ± 0.99	*p* < 0.0001	1.45	1.54 (0.93, 2.15)
SNP Tmax (sec)	165 ± 69	158 ± 52	0.54	0.19	−7 (−36.6, 22.6)
*Δ*TcpO2	3.3 ± 4.7	5.6 ± 3.6	*p* < 0.05	0.72	2.3 (0.27, 4.33)
*Δ*TcpO2_max_	10.9 ± 5.2	13.2 ± 6.9	*p* < 0.05	0.66	2.3 (−0.66, 5.26)

### Cutaneous vascular conductance

There were statistically significant differences between groups, for both endothelial-dependent (ACh) and -independent (SNP) vasodilation (Table 3). For example, healthy adults had higher baseline and maximum CVC values for ACh (e.g., 0.18 (0.09) vs 0.26 (0.09); *p* < 0.0001 and 1.39 (0.83) vs 3.52 (1.26); *p* < 0.0001 respectively). The effect size, measured by Cohen's d, was large at all instances.

On the other hand, neither ACh nor SNP Tmax reached significance [152 (66) vs 136 (59)], although for both measures, time to reach peak vascular response was lower in the healthy adults’ group. The effect size was small for both measures (e.g., 0.23 for TmaxACh and 0.19 for TmaxSNP).

## Discussion

This study compared digital microvascular responses, assessed through endothelial-dependent (ACh) and endothelial-independent (SNP) vasodilation, as well as exercise-induced oxygenation responses, between people with lcSSc and age- and gender-matched healthy adults. Building on previous work in this population (4, 5), the present findings provide a comprehensive characterisation of both resting and physiologically stimulated microvascular function, offering insight into the magnitude and functional relevance of vascular impairment in lcSSc.

Our findings demonstrate that people with lcSSc exhibit significant impairments in both endothelial-dependent and endothelial-independent vasodilatory function, alongside a reduced capacity to respond to physiological stress. Together, these findings suggest that digital microvascular dysfunction in lcSSc is not limited to endothelial abnormalities but reflects a broader impairment of vascular responsiveness. This reduced physiological reserve may contribute to impaired tissue perfusion and the clinical manifestations observed in this population, although this study was not designed to assess clinical outcomes directly.

### Endothelial-dependent function

ACh-induced vasodilation may be mediated by generation of NO, prostaglandins, or endothelial-derived hyperpolarizing factor (21, 22). Nevertheless, in people with SSc it has been demonstrated that there is an imbalance of vasodilator and vasoconstrictor mediators such as NO and endothelin, respectively, leading to an overall dysregulated vascular tone. In addition to the imbalanced vascular tone, the overall vascular dysfunction is further aggravated with the presence of cellular alterations such as large gaps between endothelial cells, the formation of vacuoles in the cytoplasm of the endothelial cell, and the damage of membrane bound storage vesicles (23). Furthermore, capillary enlargement and losses advance progressively, and intimal proliferation and proteoglycans accrue in arterioles and small arteries (24). Therefore, the imbalance in vascular tone observed in people with lcSSc compared to healthy counterparts is consistent with our findings demonstrating reduced ACh-induced vasodilatory responses in the lcSSc group.

### Endothelial-independent function

The SNP-induced vasodilation is regulated by an increase in NO activating directly the calcium-activated potassium channels (25). The decreased activation of smooth muscle cells in people with SSc compared to healthy adults could be explained by the following physiological underlying mechanisms. The vascular smooth muscle cells (VSMCs) regulate the vascular tone and pressure (26). VSMCs that are fully differentiated are expressed by a contractile phenotype and normal contractile proteins, providing a quiescent form (27). However, a certain stimulus (e.g., environmental, inflammatory, growth, or mechanical factors) could alter the contractile phenotype to a synthetic, forming thus proliferative/migratory cells and extracellular matrix component producers (26). During this phenotype alteration, the expression of alpha-smooth muscle actin and smooth muscle myosin heavy chain are deregulated, while other markers such as non-muscle myosin heavy-chain isoform-B, cellular retinol-binding protein-1, platelet-derived growth factor-A, collagen type I, and others, are newly acquired (27). This transformation has previously been described for the development of systemic hypertension and/or atherosclerosis (26, 28, 29). Interestingly, this mechanism is also present in SSc, supporting the extensive tissue fibrosis that follows the initial vasculopathy (30). These underlying physiological mechanisms may explain the reduced SNP-induced vasodilatory responses observed in people with lcSSc, further supporting the presence of a broader microvascular dysfunction extending beyond the endothelium.

### Transcutaneous oxygen pressure

Our healthy adults group presented higher *Δ*TcpO_2_ and *Δ*TcpO_2max_ values compared to people with lcSSc. TcpO_2_ measurements have been strongly correlated with blood oxygenation level dependent magnetic resonance imaging (i.e., a vascular disease assessment such as peripheral arterial occlusive disease and diabetes mellitu s^31^, ^32^) for assessing microcirculation in people with SSc (33). Thus, TcpO_2_ is considered a valid and reliable vascular assessment. Furthermore, several studies have demonstrated that chest TcpO_2_ changes at rest mimic the changes in arterial pO_2_ at rest during mild or moderate exercise (34–36) taking into consideration the relatively slow changes (90%-time response of TcpO_2_ being ∼20 s). Namely, a TcpO_2_ constant-load exercise assessment is considered accurate when is used to estimate only the absolute changes in arterial pO_2_ at rest overtime regardless of the initial starting absolute value.

The exercise-induced physiological responses include an increase in the total ventilation, and tidal volume leading to a concomitant improvement of the ventilation to perfusion ratio. As a result of these physiological responses, arterial pO_2_ is expected to rise at least for mild to moderate exercise in both normal and diseased subjects (37). Nevertheless, our findings suggest a reduced systemic microvascular and oxygenation response to physiological stress in response to combined physiological stimuli, including heat-induced and exercise-induced hyperaemia, compared to healthy adults. These findings complement, rather than directly reflect, the digital microvascular impairment demonstrated by LDF, as TcpO₂ was measured at the scapular region and therefore reflects systemic physiological responses to exercise rather than digital oxygenation. This impaired responsiveness to physiological stress is particularly relevant, as it reflects a diminished capacity of the microcirculation to adapt to stimuli that would typically promote vascular function and tissue oxygenation. Such findings provide further evidence of a compromised microvascular reserve in lcSSc.

### Clinical significance and experimental considerations

An important implication of the present findings is that the digital microcirculation in lcSSc is characterised not only by reduced baseline vasodilatory capacity, but also by a diminished responsiveness to physiological stimuli. This has direct relevance for intervention strategies, as therapeutic approaches such as exercise rely on repeated exposure to haemodynamic stimuli (e.g., shear stress and hyperaemia) to induce vascular adaptation. A blunted microvascular responsiveness may therefore limit the effectiveness of standardised exercise prescriptions in this population.

In this context, the quantification of microvascular impairment relative to healthy physiological function provides a useful framework for informing intervention design. Specifically, these findings provide a physiological rationale for investigating whether individualised exercise prescriptions may optimise microvascular adaptations in people with lcSSc. However, prospective intervention studies are required before such approaches can be recommended in clinical practice.

Furthermore, the use of non-invasive techniques such as LDF coupled with iontophoresis, alongside TcpO₂ assessment during physiological stress, may offer a valuable approach for characterising microvascular function and monitoring responses to intervention. However, whether these measures can be used to guide treatment strategies or predict responsiveness to therapy requires further investigation.

For the purposes of this study, we opted not to use invasive assessments and therefore were unable to directly examine the mechanisms underpinning the observed differences. In addition, nailfold capillaroscopy was not performed, limiting our ability to relate functional microvascular impairment to structural microangiopathy. Future studies should aim to integrate both functional and structural assessments to provide a more comprehensive understanding of vascular involvement in SSc. Several limitations should be acknowledged. Habitual physical activity was not objectively quantified, although healthy controls were recruited to be sedentary and major confounders, including smoking status and significant comorbidities, were controlled through the eligibility criteria. Furthermore, TcpO₂ was measured at the scapular region rather than the digits; therefore, these findings reflect the systemic physiological response to exercise and should not be interpreted as direct measures of digital oxygenation. Although participants with lcSSc were on stable medical therapy, including vasoactive medications, for at least 8 weeks prior to testing, the potential influence of pharmacological treatment on microvascular function cannot be completely excluded. Blinding of the assessor was not feasible owing to the visible clinical manifestations of lcSSc; however, the use of objective instrument-derived outcomes and standardised testing procedures minimised the potential for observer bias. Finally, the inclusion of only people with lcSSc may limit the generalisability of the findings to the broader SSc population, although it enabled a more homogeneous cohort and reduced variability related to disease severity and organ involvement.

## Conclusion

People with lcSSc demonstrate significant impairment in digital microvascular function at rest together with a reduced systemic physiological response to exercise compared with age- and sex-matched healthy adults. These findings highlight impaired microvascular physiological reserve and provide a mechanistic rationale for future studies investigating whether individualised exercise interventions can improve vascular function and clinically meaningful outcomes in this population. Quantifying the magnitude of this impairment relative to healthy function provides important insight into the vascular limitations present in lcSSc and establishes a physiological basis for future studies investigating targeted and individualised exercise interventions. Further research is required to determine how these physiological impairments relate to clinically meaningful outcomes and responsiveness to exercise interventions.

## Data Availability

The raw data supporting the conclusions of this article will be made available by the authors, without undue reservation.
